# Validity of the Modified Baecke Questionnaire: comparison with energy expenditure according to the doubly labeled water method

**DOI:** 10.1186/1479-5868-5-30

**Published:** 2008-05-27

**Authors:** Emmy M Hertogh, Evelyn M Monninkhof, Evert G Schouten, Petra HM Peeters, Albertine J Schuit

**Affiliations:** 1 Julius Center for Health Sciences and Primary Care, University Medical Center Utrecht, PO Box 85500, 3508 GA, Utrecht, The Netherlands; 2 Division of Human Nutrition, Wageningen University, Wageningen, The Netherlands; 3 National Institute of Public Health and the Environment, Bilthoven, The Netherlands; 4 Institute of Health Science, Vrije Universiteit Amsterdam, The Netherlands

## Abstract

**Background:**

In epidemiological research, physical activity is usually assessed by questionnaires. Questionnaires are suitable for large study populations since they are relatively inexpensive and not very time consuming. However, questionnaire information is by definition subjective and prone to recall bias, especially among elderly subjects. The Modified Baecke Questionnaire, developed by Voorrips and coworkers, measures habitual physical activity in the elderly. The questionnaire includes questions on household activities, sports, and leisure time activities, over a time period of one year. The Modified Baecke Questionnaire results in a score to classify people as high, moderate, or low in daily physical activity, based on tertiles.

**Methods:**

The validity of the Modified Baecke Questionnaire score was assessed among 21 elderly men and women using the doubly labeled water method as the reference criterion. This method is considered to be the gold standard for measuring energy expenditure in free-living individuals. Energy expenditure on physical activity is estimated by the ratio of total energy expenditure measured by the doubly labeled water method and resting metabolic rate measured by indirect calorimetry. This ratio is called the physical activity ratio.

**Results:**

The Spearman correlation coefficient between the questionnaire score and the physical activity ratio (PAR) was 0.54 (95% CI 0.22–0.66). Correct classification by the questionnaire occurred in 71% of participants who were in the lowest tertile of PAR, in 14% of participants in the middle tertile, and in 43% of participants in the highest tertile. Subjects were not wrongly classified in an opposite tertile.

**Conclusion:**

The validity of the Modified Baecke Questionnaire is fair-to-moderate. This study shows that the questionnaire can correctly classify individuals as low or high active, but does a poor job for moderately active individuals.

## Background

Physical activity is known to have a positive effect on health and contributes to developing and maintaining a high quality of life [1,2]. In epidemiological research, physical activity is usually assessed by questionnaires. Questionnaires are suitable for large study populations since they are relatively inexpensive and not very time consuming for the study participants. However, questionnaires are subjective methods, where participants can easily overestimate or underestimate their time spent on activities. Especially in elderly persons, questionnaires may be prone to recall problems, since these persons may suffer from impaired memory. Only a few physical activity questionnaires have been validated in elderly persons using the doubly labeled water (DLW) method as reference [3-6].

The DLW method is considered to be the gold standard for measuring energy expenditure in free-living individuals and has shown to be valid for human use [7-9]. This noninvasive method allows subjects to maintain their habitual activities and causes only minimal inconvenience. However, the DLW method is relatively expensive and time-consuming, what makes it unsuitable for use in large population studies. Because of its accuracy, it is however valid for use in smaller subsets of populations for validation purposes of other, more easy applicable, measurement techniques such as questionnaires [10].

Baecke and coworkers developed a short questionnaire to measure physical activity in healthy persons by referring to activities over the past year [11]. This questionnaire was slightly modified (Modified Baecke Questionnaire) by Voorrips and coworkers, to capture habitual physical activity specific in the elderly [12]. It includes questions about household activities, sports, and leisure time activities and results in a continuous score.

The Modified Baecke Questionnaire is a frequently used questionnaire to measure habitual physical activity in the elderly, over the past year. Earlier validation of the questionnaire score with doubly labeled water resulted in a low validity (Spearman correlation coefficient of 0.14) [3]. We believe this was the result of a methodological shortcoming in the previous study, because ten questionnaires were simultaneously validated, with the risk of possible interference. Furthermore, the interviewers reminded the subjects about activities when inconsistent answers between questionnaires were given.

To assess the validity of the Modified Baecke Questionnaire score, we compared the results of this questionnaire with the results obtained from the DLW method.

## Methods

### Subjects

Subjects for the validation study were recruited among participants of a randomized controlled intervention study that investigated the effect of a six-month training programme on several cardiovascular risk factors [13]. Participants were elderly men and women, aged 60 to 80 years, living in Arnhem, a middle-sized city in the Netherlands. The training programme consisted of three group sessions a week of 45 minutes performing aerobic exercise, calisthenics, and flexibility exercises. Exclusion criteria were: heart failure, angina pectoris, insulin dependent diabetes mellitus, use of beta-blockers, and myocardial infarction or stroke in the preceding two years. Subjects taking diuretic drugs were excluded since these drugs might disturb DLW measurements by influencing hydration.

Participants of the intervention study were stratified in tertiles of physical activity levels based on the Physical Activity Scale for the Elderly (PASE) [14]. Out of these tertiles, a total of 22 subjects was selected equally divided over intervention and control group. Thus a broad range of activities could be expected. Before the start of the validation study, one man dropped out, leaving 10 men and 11 women in the validation study. The study was approved by the medical ethics committee of the Wageningen Agricultural University and all subjects gave their written informed consent.

### Doubly labeled water method

The DLW method estimates total energy expenditure (TEE) in free living subjects. Subjects receive a weighted amount of water labeled with an isotope of hydrogen (^2^H, deuterium) and an isotope of oxygen (^18^O). The ^2^H is excreted from the body as water; the ^18^O is excreted as water and carbon dioxide. The subsequent excretion in urine of these isotopes is measured by mass spectrometry. The difference between the excretion rates is proportional to the carbon dioxide production and hence energy expenditure.

Since energy expenditure on physical activity is difficult to measure, it is usually estimated by calculating the ratio of TEE and resting metabolic rate (RMR). This ratio is known as the physical activity ratio (PAR).

In this validation study in 21 subjects, TEE was measured over a 2-week period with the DLW method. At the beginning and at the end of the 2-week period, RMR was measured with a ventilated hood (indirect calorimetry). The Modified Baecke Questionnaire was administered at the end of the DLW period.

### Measurements

#### The Modified Baecke Questionnaire

The Modified Baecke Questionnaire was developed to measure habitual physical activity in the elderly. The questionnaire includes items about household activities, sport, and leisure time activities over the past year. The Modified Baecke Questionnaire is an adapted version of the physical activity questionnaire of Baecke and coworkers. Additional questions about household activities were included to replace the original questions about occupation to make the questionnaire applicable for use in elderly subjects. The questions on household activities have four to five possible answers, classifying the activity from inactive to very active. Questions about sport and leisure time activities include the type of activity, the frequency of performance, and the number of months per year that the activity is performed. All items result in a separate score that incorporates activity duration, frequency, and an intensity code based on energy costs. Summing the household score, sport score, and leisure time activity score results in a continuous overall unitless activity score. In this study, tertiles were computed that classified people as low, moderate or high physically active. The questionnaire was administered as a face to face interview, by an experienced research assistant.

#### Total energy expenditure by the doubly labeled water method

The DLW method was used to measure TEE for a period of two weeks, using the Maastricht protocol [15]. Subjects received a weighted amount of labeled water containing 5 At% ^2^H_2_O and 10 At% H_2_^18^O, calculated to raise the baseline ^18^O and ^2^H level at least 300 ppm and 150 ppm, respectively. DLW was administered after collecting a background urine sample between 09.00 pm and 11.00 pm at home. The solution was ingested and the container was washed with tap water that was also ingested by the subject. Subsequently, urine was collected the next day, after 1 week, and after 2 weeks. Samples were taken from a second voiding early in the morning and between 09.00 pm and 11.00 pm. Urine samples were stored at -20°C and analyzed with isotope ratio mass spectrometry (Aqua Sira, VG, UK). The ratio between the deuterium dilution space (Nh) and oxygen-18 dilution space (No) was 1.032 ± 0.006 (mean ± SD), range 1.015–1.044.

#### Resting metabolic rate

RMR was measured twice, at the start and at the end of the 2-week DLW period. RMR was measured by indirect calorimetry, using an open-circuit ventilated hood system. Periodical alcohol combustion was used as a reference to which all measurements were standardized. Measurements were done under standard conditions, with the person lying half supine in a comfortable warm room (21°C), watching non-stressing movies, and having fasted for 12 hours. The subject came to the health centre by car, avoiding extra physical exercise. Although measurements continued for 60 minutes, the mean energy expenditure of the last 45 minutes was used. The mean of the two RMR measurements was used for data analysis.

#### Body composition

Weight (kg) and height (m) were measured at the beginning and end of the study period. Weight was measured to the nearest 0.05 kg using a calibrated digital scale (ED60-T, Berkel, Rotterdam, The Netherlands), after voiding, with subjects wearing only light underwear. Height was measured with a wall-mounted stadiometer, to the nearest 0.5 cm. Body mass index was calculated as body weight divided by height squared.

### Statistical analysis

Mean values of age, body mass index, RMR, TEE, and PAR were calculated for men and women, separately. The total Modified Baecke Questionnaire score and subscores were calculated for all subjects. The distribution of the total score and subscores was skewed, and therefore median values were reported. Possible gender differences were determined by a t-test or Mann-Whitney test for parametric and nonparametric data, respectively.

Validity of the Modified Baecke questionnaire score was studied by comparing the score with the PAR using Spearman correlation coefficients. Confidence intervals were computed by bootstrapping techniques [16]. Two thousand samples were taken to determine Spearman correlation coefficients. All correlation coefficients were sorted in an ascending way, and the 50^th ^and 1950^th ^coefficient were taken as borders of the 95% confidence interval. In addition, the mean PAR was calculated for tertiles of the questionnaire score and Tukey's test was performed to investigate differences between the tertiles.

All analyses were completed using SAS version 9.1.

## Results

Table 1 summarizes the characteristics of the 21 study participants, stratified by sex. Mean age of the population was 69.9 years and mean BMI 25.2 kg/m^2^. There were statistically significant gender differences for RMR and TEE, with men having a higher score than women. There was a large variation in physical activity level in this group of elderly persons. PAR ranged from 1.40 to 2.22. Questionnaire scores ranged from 2.3 to 35.7. Men showed a statistically significant lower score on household activities compared to women.

**Table 1:** Baseline characteristics of the study population

	**Men (n = 10)**	**Women (n = 11)**	**Total (n = 21)**
	
	**Mean (SD)**	**Mean (SD)**	**Mean (SD)**
Age (years)	70.6 (3.8)	69.2 (4.8)	69.9 (4.3)
Body mass index (kg/m^2^)	26.3 (2.2)	24.3 (3.1)	25.2 (2.8)
RMR ^# ^(MJ/day)	6.8 (0.8)^a^	5.2 (0.7)	6.0 (1.1)
TEE ^&^ (MJ/day)	12.0 (1.4)^a^	9.6 (0.6)	10.7 (1.6)
PAR *	1.78 (0.22)	1.85 (0.23)	1.82 (0.22)
	**Median (range)**	**Median (range)**	**Median (range)**
Modified Baecke Questionnaire score	11.9 (5.6–23.2)	14.2 (2.3–35.7)	13.7 (2.3–35.7)
- Household score	1.6 (1.4–2.3)^a^	2.4 (1.0–2.8)	1.9 (1.0–2.8)
- Sport score	2.2 (0.0–9.7)	2.1 (0.0–11.3)	2.1 (0.0–11.3)
- Leisure score	5.7 (0.7–16.8)	7.0 (0.0–30.8)	6.1 (0.0–30.8)

# RMR = Resting metabolic rate, measured by indirect calorimetry

& TEE = Total energy expenditure, measured by the doubly labeled water method

* PAR = Physical activity ratio, ratio of TEE and RMR

^a ^Significantly different from women

**Figure 1:**
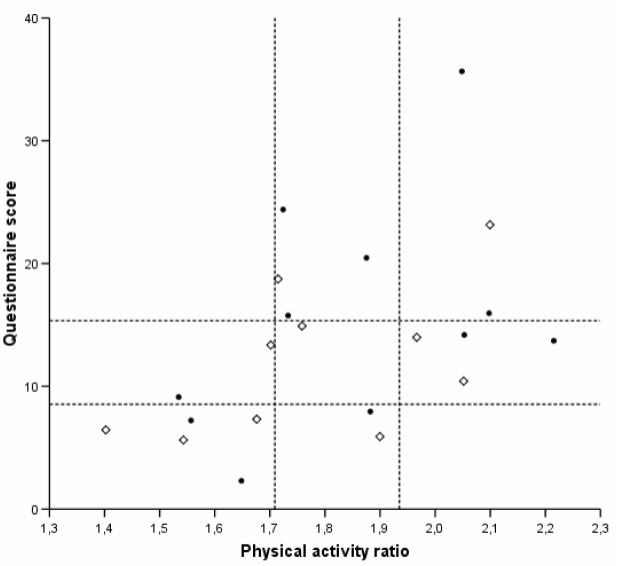
**Association between the Modified Baecke Questionnaire score and the physical activity ratio (r = 0.54, 95% CI 0.22–0.66)**. Diamonds are men, circles are women. Lines represent the tertile borders. Physical activity ratio (PAR) is the ratio of total energy expenditure and resting metabolic rate.

Figure 1 shows the correlation between the questionnaire score and PAR. The overall Spearman correlation coefficient was 0.54 (95% CI 0.22–0.66). For men it was 0.56 (95% CI -0.03–0.86), for women 0.43 (95% CI 0.00–0.71).

Table 2 shows the mean PAR for the tertiles of the questionnaire score. Mean PAR did not differ significantly between the tertiles of the questionnaire score, when using Tukey's test.

**Table 2:** Mean PAR per tertile of Modified Baecke Questionnaire score

	**Tertile of Modified Baecke Questionnaire score**		
	
	**Lowest (range 2.30–7.94)**	**Middle (range 9.13–14.91)**	**Highest (range 15.77–35.65)**
PAR *	1.66 (1.49–1.83)	1.90 (1.68–2.12)	1.90 (1.73–2.07)

* PAR = Physical activity ratio, ratio of TEE and RMR, PAR is presented as mean and 95% CI

As shown in Figure 1, of the participants who were in the lowest tertile of PAR, 5/7 (71%) were correctly classified as low active by the questionnaire. For participants in the middle tertile 1/7 (14%) were correctly classified by questionnaire and for those in the highest tertile 3/7 (43%) were correctly classified by the questionnaire. Large misclassification, i.e. classification of subjects into the lowest tertile on the questionnaire score and into the highest tertile on the PAR or the other way around, did not occur.

## Discussion

The Modified Baecke Questionnaire is designed to measure habitual physical activity in the elderly. Correlation with the DLW method as a reference method, showed fair-to-moderate correlation (Spearman correlation coefficient 0.54). This study shows that the questionnaire does a fairly good job of classifying individuals as low active and high active, but poorly for moderately active.

In the past ten years, several physical activity questionnaires have been validated using the DLW method [3-6,17-23]. Published correlation coefficients varied widely between the different questionnaires. Also studies investigating the validity of the same questionnaire in different populations resulted in dissimilar correlation coefficients, indicating that one should be cautious when generalizing results to populations with different characteristics.

The Modified Baecke Questionnaire has been validated before by Bonnefoy and coworkers, also using the DLW method [3]. In a healthy population of 19 older men, the Spearman correlation coefficient between the PAR and the Modified Baecke Questionnaire score was 0.14. This poor result is probably due to the way in which the questionnaire was administered. First of all, the study simultaneously validated ten questionnaires, which were completed during a personal interview of 4 hours and, therefore, the questionnaires might have influenced each other. In our study, we also have this potential bias, although we only administered one more questionnaire (PASE) during the interview, instead of nine. Moreover, in the study of Bonnefoy and coworkers, the bias might also be greater since the interviewers reminded the subjects about activities when inconsistent answers between the questionnaires were given. This was not done in our study.

Other physical activity questionnaires that are especially developed for administration in elderly subjects and have been validated using the DLW method are the Physical Activity Scale for the Elderly (PASE) [14], the Yale Physical Activity Survey (YPAS) [24], and the Zutphen Physical Activity Questionnaire [25].

Validation of the PASE with DLW has been performed in the same study population as the current study [4]. The Spearman correlation coefficient between the PASE score and PAR was 0.68 (95% CI 0.35–0.86). The higher correlation coefficient found for the PASE score than for the Modified Baecke Questionnaire score might be explained by the difference in period over which physical activity was assessed. The PASE contains questions referring to activities during the past week and fully covered the period when the DLW measurement was conducted, whereas the Modified Baecke Questionnaire comprises questions referring to activities in the last year. Due to the incongruence of time frames, the correlation can be diluted. However, the DLW method is still the reference standard to measure energy expenditure and it is not feasible to conduct the measurement over a long period.

The validity of the Zutphen Physical Activity Questionnaire score was somewhat higher than for the Modified Baecke Questionnaire score (Spearman correlation coefficient of 0.67, p < 0.01) [26]. It should be noted that this study was performed in a population of only elderly men.

The PASE and YPAS have also been validated by Bonnefoy and coworkers. The Spearman correlation coefficients between PAR and the questionnaire scores were 0.24 and 0.03, respectively. These validation results should be considered with caution because of above mentioned limitations in the conduct of that study.

Strength of our study is that the selection of subjects was based on tertiles of the PASE score. Hence all levels of physical activity were represented in the study population. Because participants were enrolled in an exercise intervention study and therefore where more aware of their physical activity behavior, they would likely to be able to more accurately report their level of activity. This should be taken into account when generalizing the correlation results to the general population. Our study has also several limitations that have to be addressed. First, the small study population restricted the possibility to make valid estimates of the validity of the questionnaire in general, but especially about the validity of the questionnaire for men and women separately. Second, the PASE questionnaire was administered before the Modified Baecke Questionnaire, which might have influenced the validation results slightly.

## Conclusion

The validity of the Modified Baecke Questionnaire is fair-to-moderate. This study shows that the questionnaire does a fairly good job of classifying individuals as low active and high active, but poorly for moderately active.

## Competing interests

The authors declare that they have no competing interests.

## Authors' contributions

EGS is the supervisor of the study, AJS conducted the study measurements, EMH performed the statistical analyses, EMH, EMM, PHMP, and AJS drafted the manuscript. All authors read and approved the final manuscript.
